# Supplementary material for: Optimizing future biodiversity sampling by citizen scientists
Source: Proc Biol Sci. 2019 Oct 2;286(1912):20191487. doi: 10.1098/rspb.2019.1487 (PMC6790778; doi:10.1098/rspb.2019.1487)
Supplement: Appendices 1-5 [file rspb20191487supp1.pdf]

Electronic Supplementary Material for:

Callaghan, C. T.*, Poore, A. G. B., Major, R. E., Rowley, J. J. L., and Cornwell, W. K. 2019. Optimizing future biodiversity sampling by citizen scientists. Proceedings of the Royal Society B. https://doi.org/10.1098/rspb.2019.1487

*Corresponding author:
Corey T. Callaghan
Centre for Ecosystem Science
School of Biological, Earth and Environmental Sciences
UNSW Sydney
E: c.callaghan@unsw.edu.au
P: +61 421 601 388

Appendix 1. Specific predictions of biodiversity sampling in space and time, which we tested after assigning every citizen science observation a measure of marginal value.

Table S1. The predictions of biodiversity sampling in space and time, and a brief description of each.

Prediction	Description
**Site sampling**	If a site has been previously sampled or not. We predicted that unsampled sites would be marginally more valuable than previously sampled sites.
**Median sampling interval**	The median of the distribution of waiting times between samples at a site. We predicted that the median sampling interval would be positively associated with the value of a citizen science observation; i.e., observations from sites with high median waiting times would be more valuable than observations from sites with low median waiting times.
**Days since last sample**	The number of days between samples at a site. We predicted that the number of days since the last sample would be positively associated with the value of a citizen science observation.
**Distance to the nearest sampled site**	The distance between the site in question and the nearest sampled site. We predicted that the distance to the nearest sampled site would be positively associated with the value of a citizen science observation.
**Nearest neighbor sampling interval**	The median sampling interval of the nearest neighbor. We predicted that the nearest-neighbor sampling interval would positively influence the value of an observation, whereby well-sampled areas (i.e., multiple sites near each other with low median sampling intervals) would have lower value.
**Number of unique days sampled**	The total number of unique days sampled for a given site. We predicted that the total number of unique days would be positively associated with the value of an observation, whereby sites with many observations would have additional value given the long-term data originating from them.

Appendix 2. A sensitivity analysis of trend estimates for the top 50 species included in our analysis.

We performed a sensitivity/power analysis to investigate the robustness of our population trend models and to identify any critical thresholds which exist to provide inferences of population trends. We randomly subsampled the potential pool of eBird checklists (N=25,995) from 10% to 100% of the checklists, in 5% increments. We performed 100 runs of the same GLM as presented in the main manuscript, at each of these 19 levels, for each of the top 50 species; fitting a total of 95,000 models. We removed any models that did not converge and whose slope estimates were 2 SD > the mean and 2 SD < mean. We then plotted the percent of sampled eBird checklists against the slope estimate for the continuous day model parameter (i.e., the trend slope estimate), to investigate whether or not these slope estimates converged.

Figures S1a – S1e demonstrate that the slope estimates do converge, but at largely different sample sizes (cf. Sulphur-crested Cockatoo versus Welcome Swallow). They also show varying ranges in slope estimates among species. Fig S2 shows the degree to which the range of slope estimates varies among species, by plotting the max range (i.e., the maximum difference in any two slope estimates for a given species) for each species. Pied Currawong had the lowest range in slope estimates and Galah had the largest range in slope estimates.

We further investigated this by making the y-axis 'unitless'. To do this, we first calculated the 'best estimate' – e.g., the average slope estimate when using the maximum amount of data for each species, and then calculated the relaitve value of the difference from that estimate as a function of sample size (i.e., percent of the possible checklists).

Figures S3a – S3e clearly show that the relative difference from the best estimate again varies among species, with some species not needing a large additional pool of eBird checklists to approach the best estimate (e.g., Pied Currawong) and other species needing more eBird checklists (e.g., Australian Raven). Visual inspection shows different critical thresholds for different species (cf., Welcome Swallow and Rainbow Lorikeet). To quantify this, we calculated when the maximum absolute difference was reduced by at least 50% – a rough measure of convergence of the absolute difference – for each species, and calculated at what sampling level (i.e., number of eBird checklists) this occurred.

We found that the median number of checklists necessary for the convergence of slope estimates (Fig. S4) to 50% reduction in the absolute difference between the best estimate was ~11,700, with a min of ~ 6,500 and a max of ~ 20,800. We note however, that these analyses are preliminary, and future work should investigate these questions further.

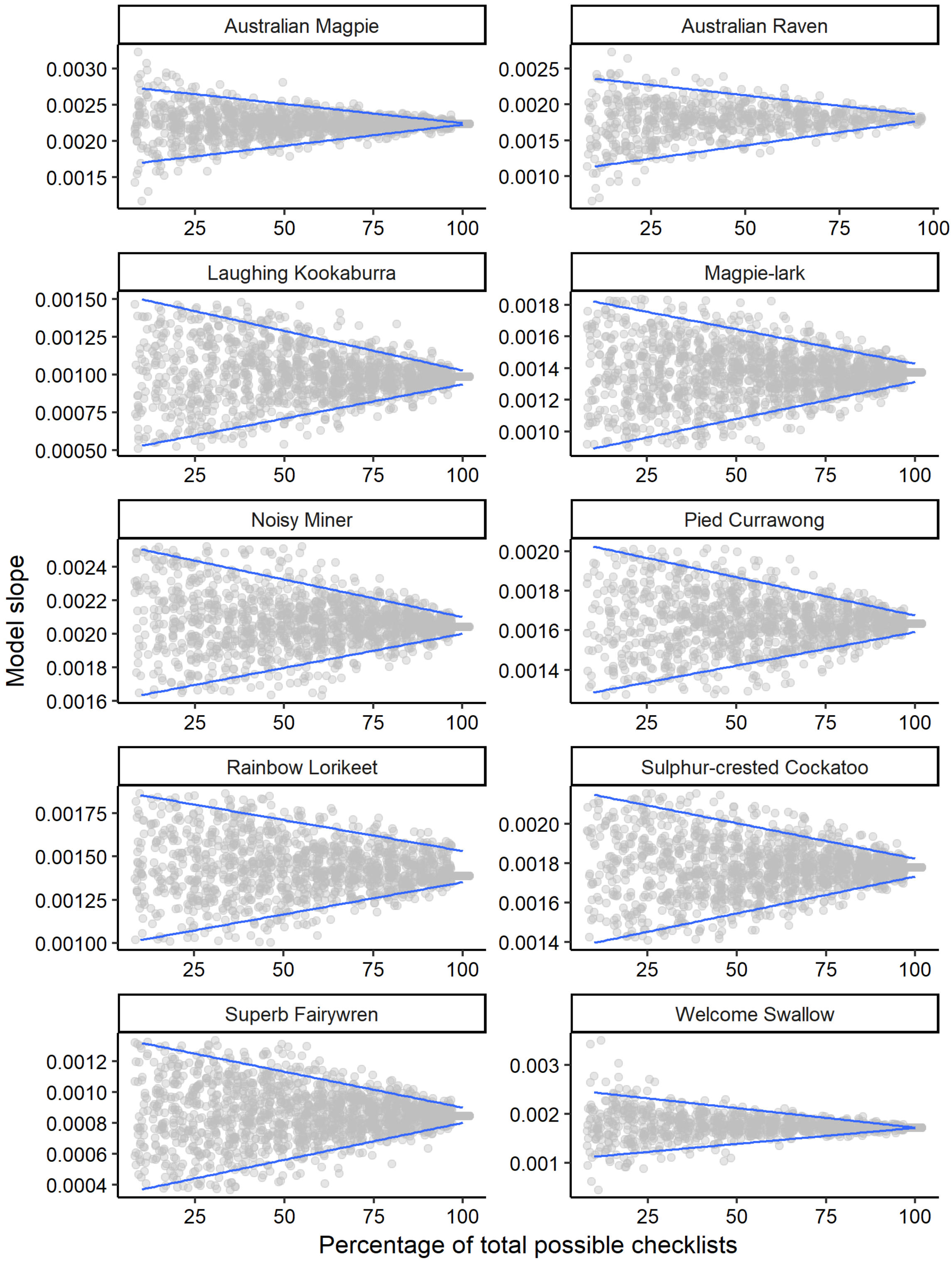

Fig S1a. The first ten species for which we plotted the trend estimate as a function of the percentage of total possible eBird checklists. The blue lines represent a quantile regression with 5 and 95% confidence limits. Note that the y-axis is different among species.

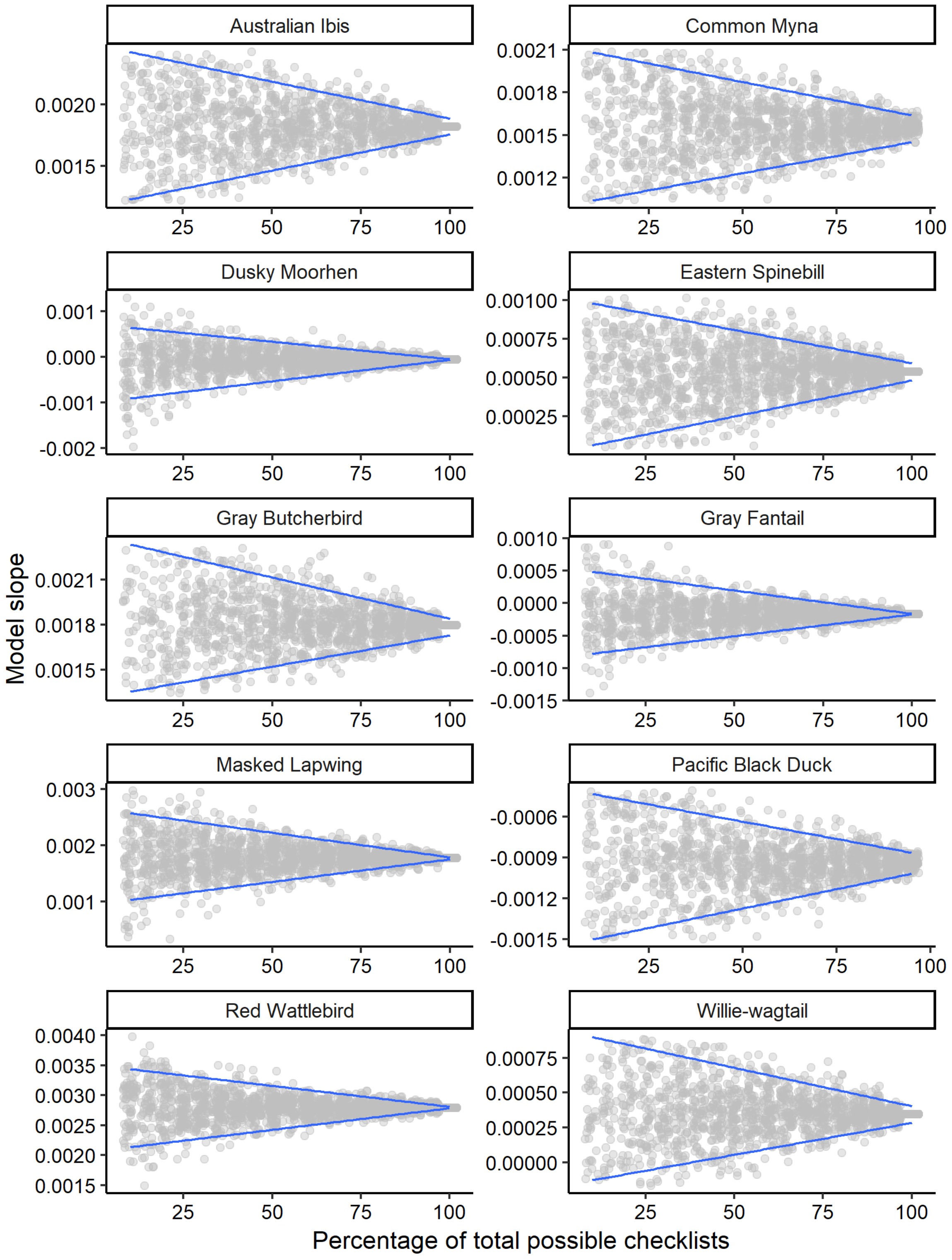

Fig S1b. The 11th – 20th species for which we plotted the trend estimate as a function of the percentage of total possible eBird checklists. The blue lines represent a quantile regression with 5 and 95% confidence limits. Note that the y-axis is different among species.

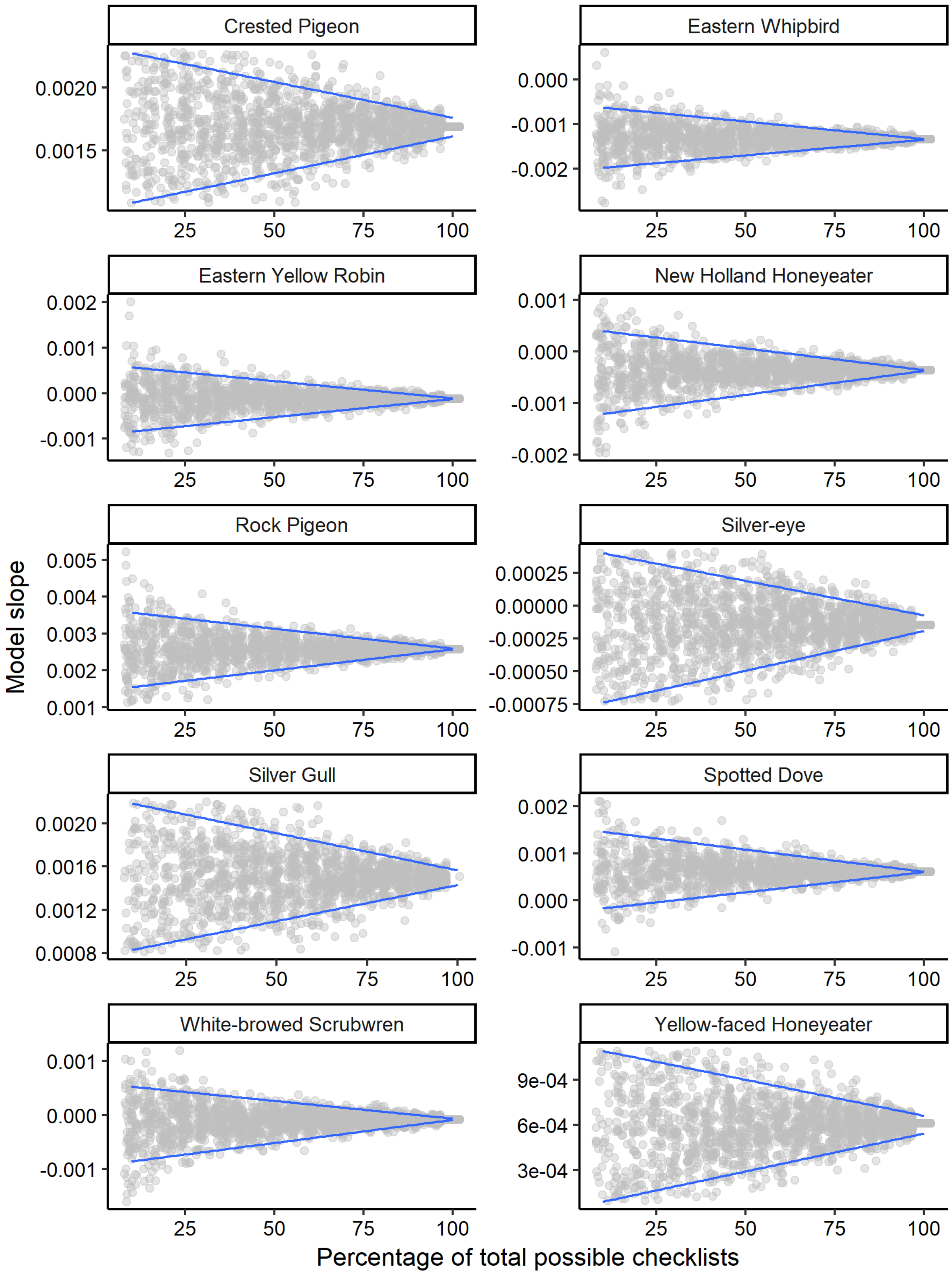

Fig S1c. The 21st – 30th species for which we plotted the trend estimate as a function of the percentage of total possible eBird checklists. The blue lines represent a quantile regression with 5 and 95% confidence limits. Note that the y-axis is different among species.

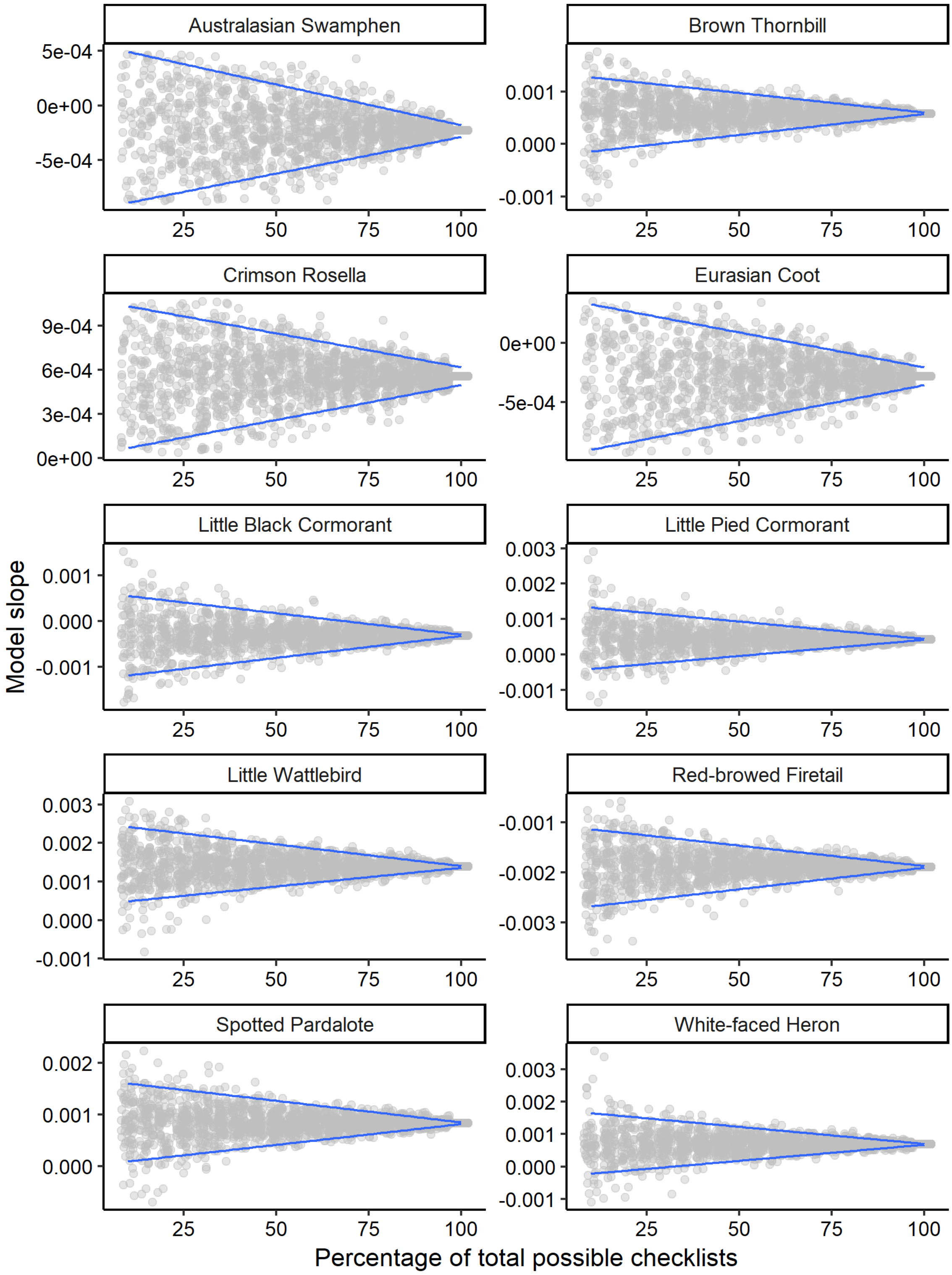

Fig S1d. The 31st – 40th species for which we plotted the trend estimate as a function of the percentage of total possible eBird checklists. The blue lines represent a quantile regression with 5 and 95% confidence limits. Note that the y-axis is different among species.

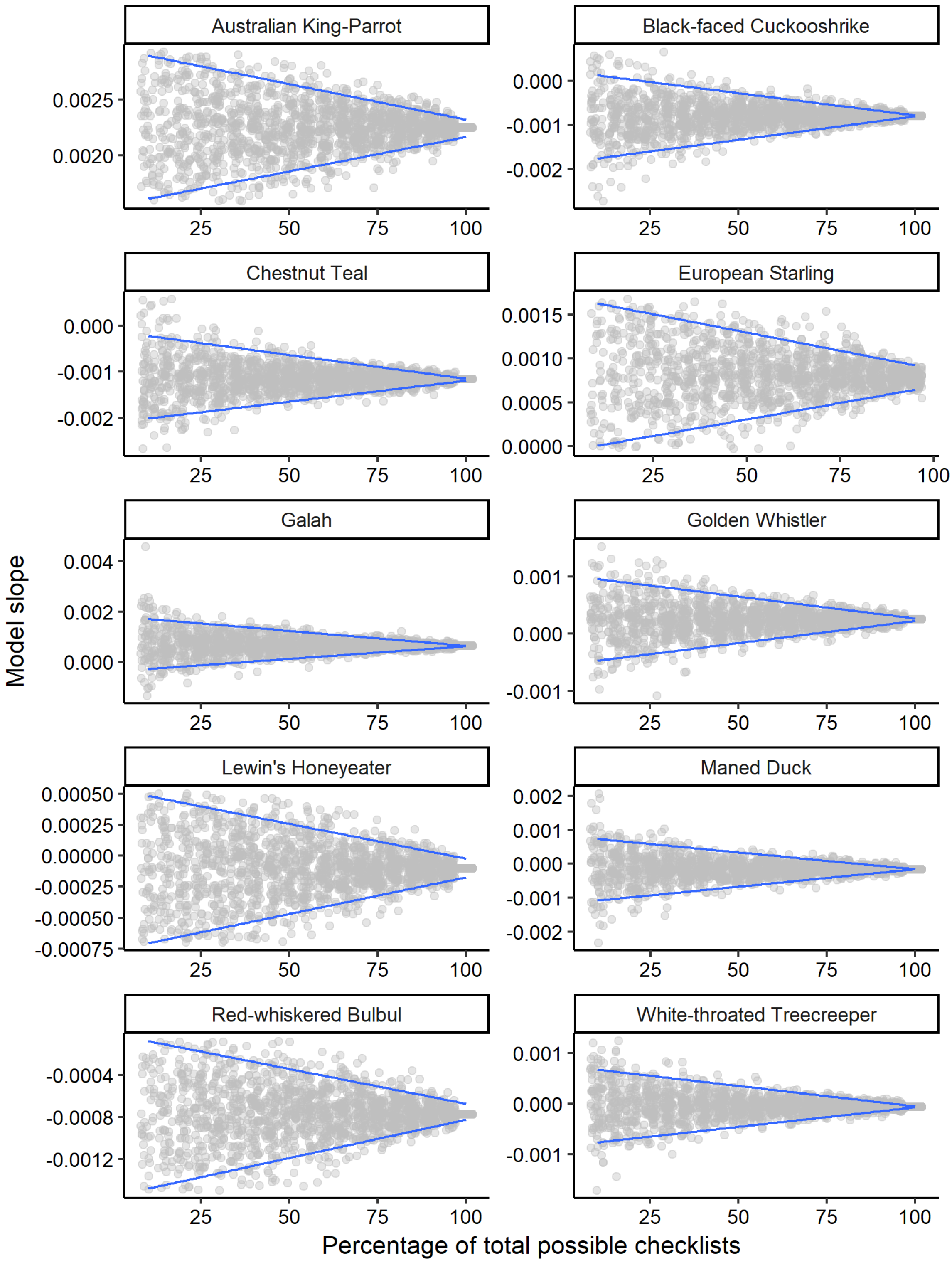

Fig S1e. The 41st – 50th species for which we plotted the trend estimate as a function of the percentage of total possible eBird checklists. The blue lines represent a quantile regression with 5 and 95% confidence limits. Note that the y-axis is different among species.

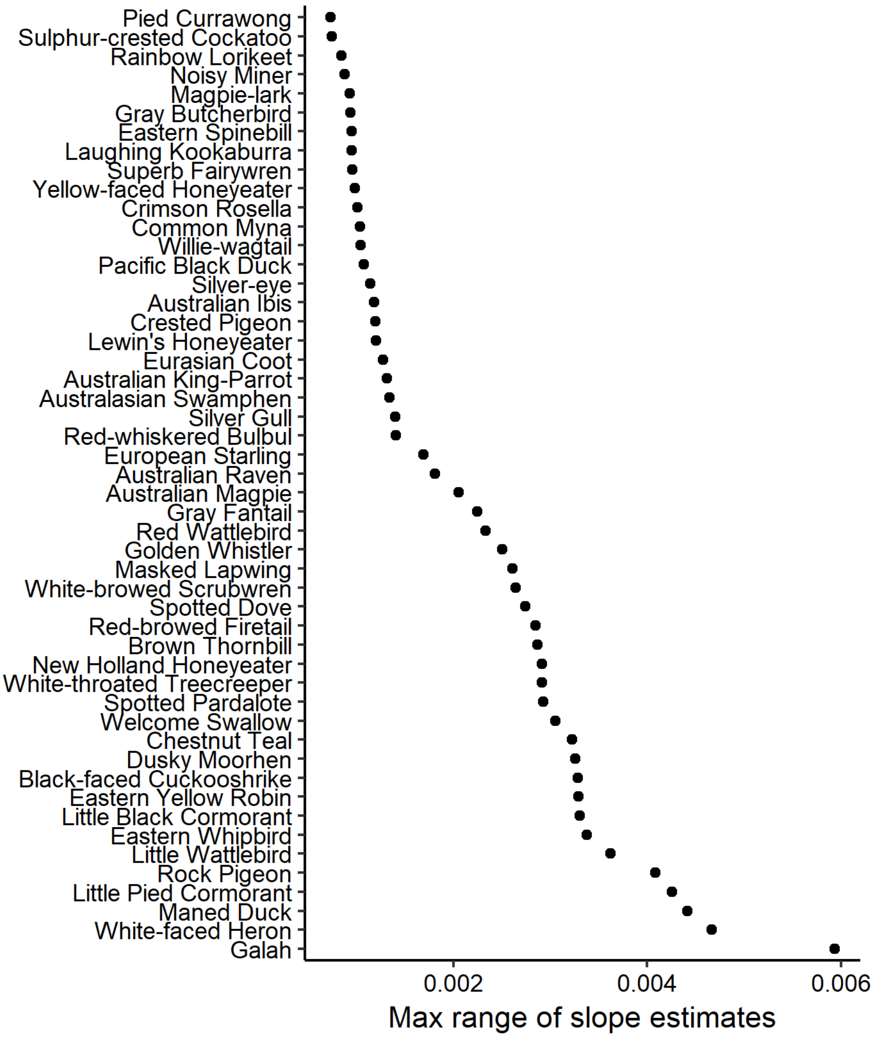

Fig. S2. The max range (i.e., the maximum difference in any two slope estimates for a given species) for each species. Pied Currawong had the lowest range and Galah had the largest range.

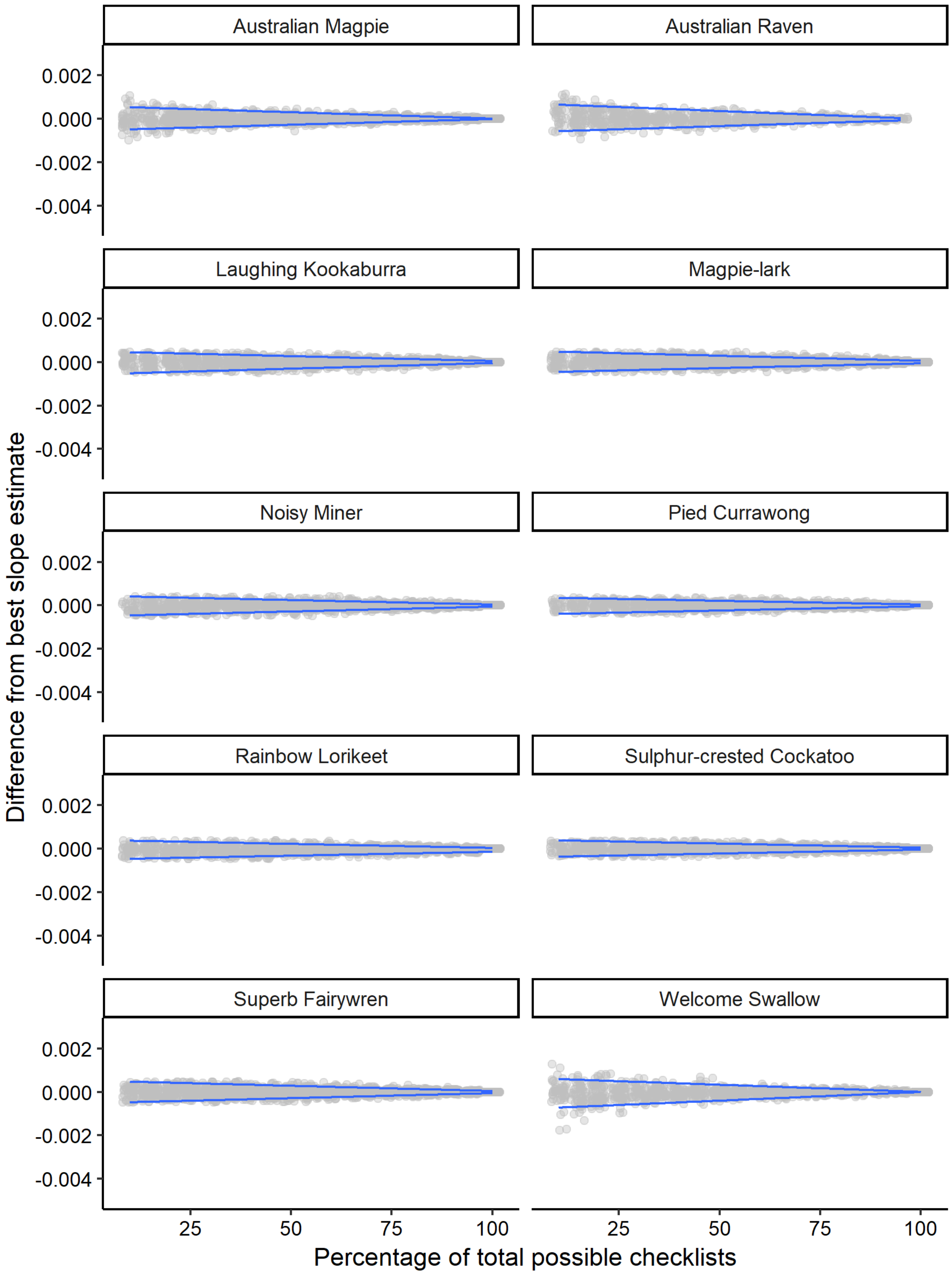

Fig. S3a. The relative difference from the best slope estimate for each species (i.e., the mean slope estimate calculated when using all available data), shown as a function of the percentage of total possible checklists. The blue lines represent a quantile regression with 5 and 95% confidence limits. Note that the y-axis is the same among species.

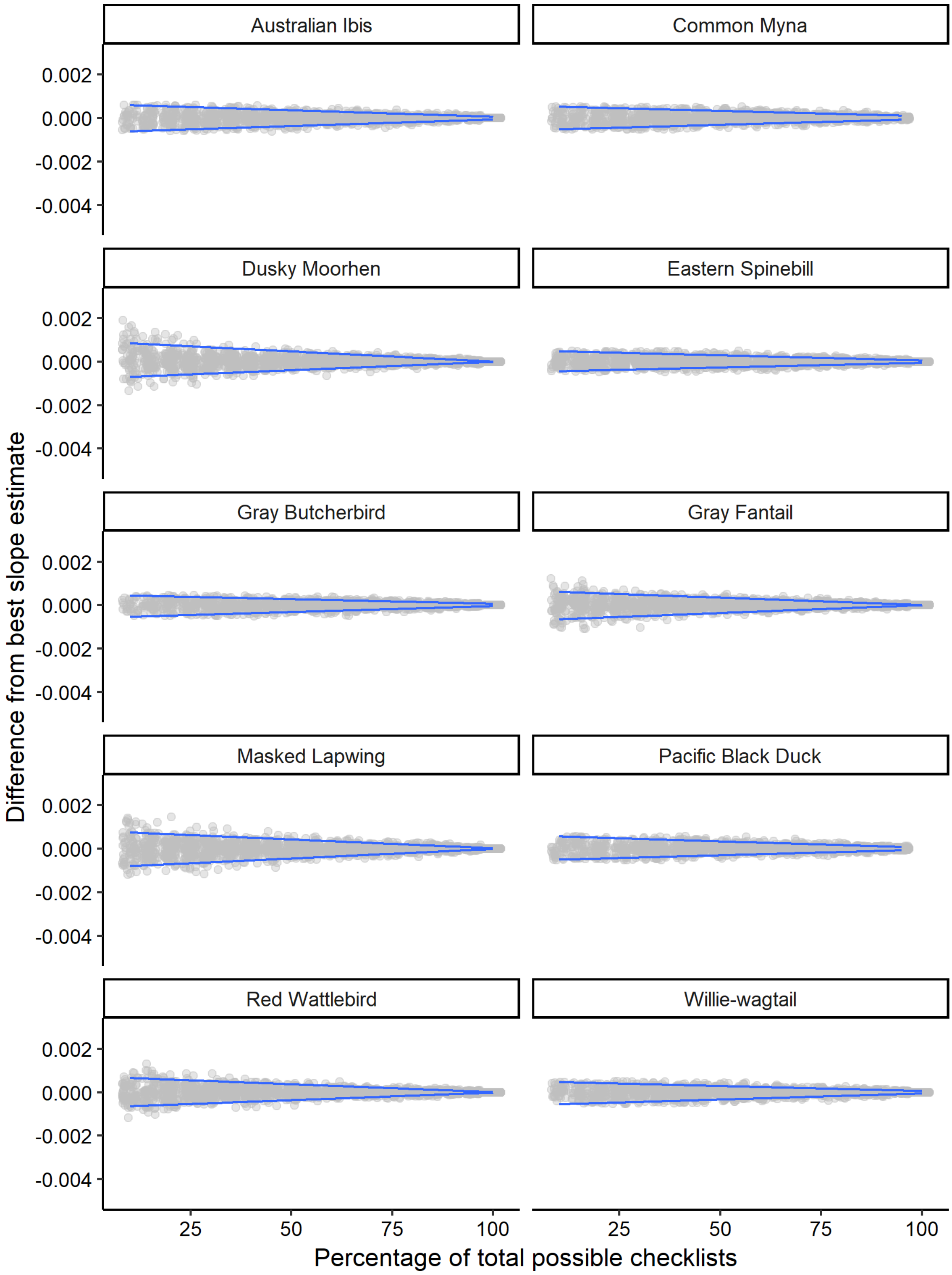

Fig. S3b. The relative difference from the best slope estimate for each species (i.e., the mean slope estimate calculated when using all available data), shown as a function of the percentage of total possible checklists. The blue lines represent a quantile regression with 5 and 95% confidence limits. Note that the y-axis is the same among species.

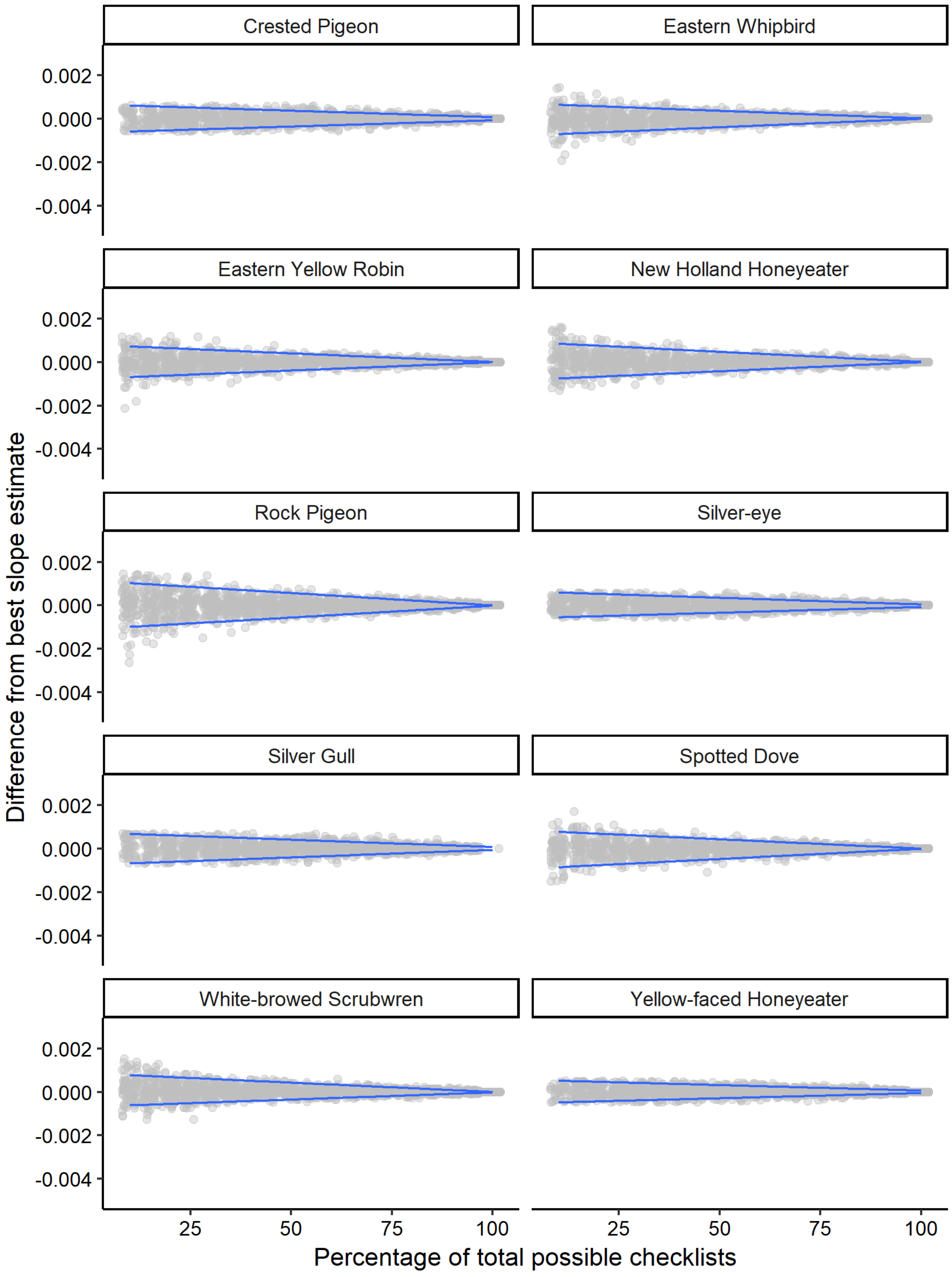

Fig. S3c. The relative difference from the best slope estimate for each species (i.e., the mean slope estimate calculated when using all available data), shown as a function of the percentage of total possible checklists. The blue lines represent a quantile regression with 5 and 95% confidence limits. Note that the y-axis is the same among species.

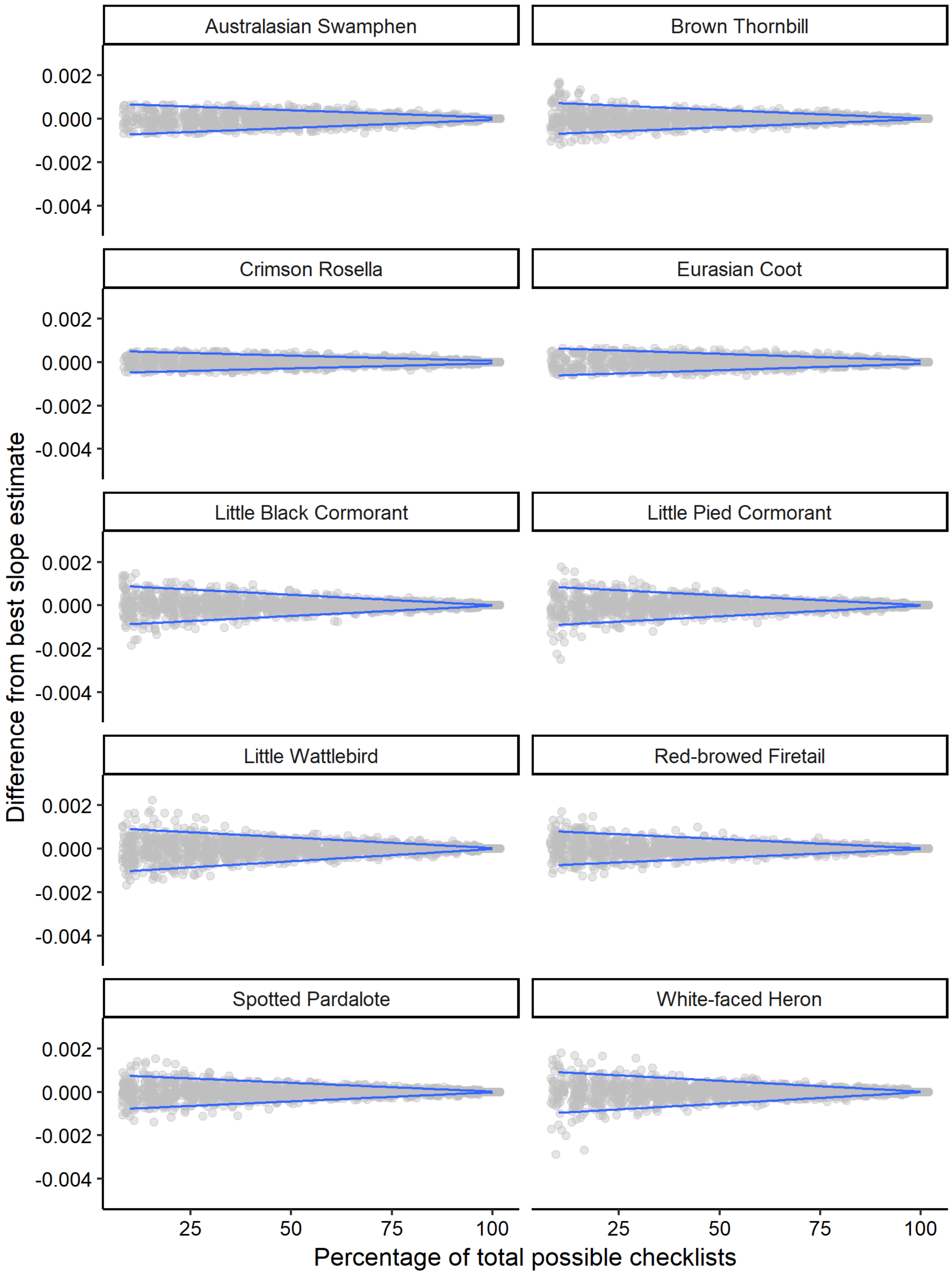

Fig. S3d. The relative difference from the best slope estimate for each species (i.e., the mean slope estimate calculated when using all available data), shown as a function of the percentage of total possible checklists. The blue lines represent a quantile regression with 5 and 95% confidence limits. Note that the y-axis is the same among species.

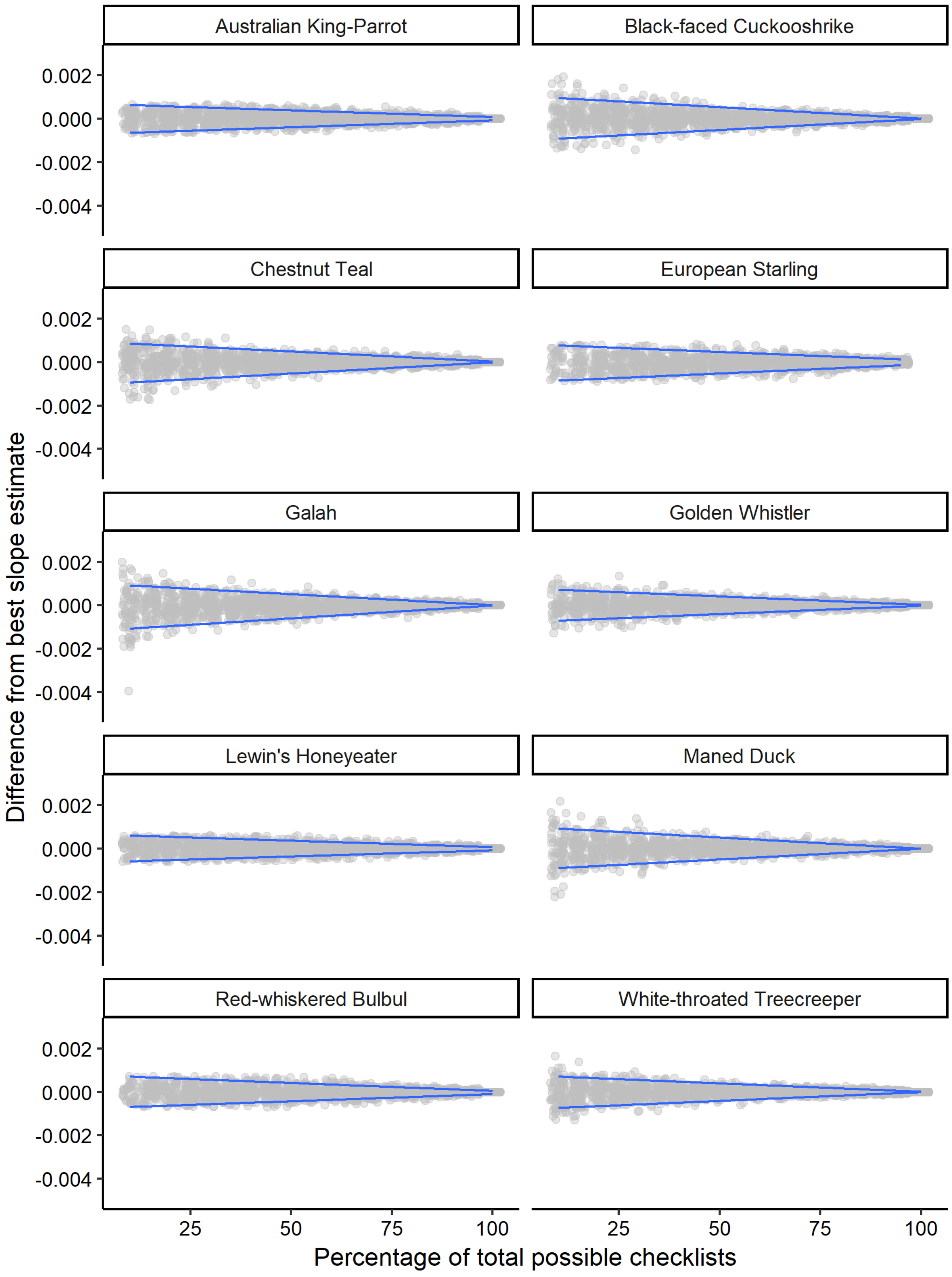

Fig. S3e. The relative difference from the best slope estimate for each species (i.e., the mean slope estimate calculated when using all available data), shown as a function of the percentage of total possible checklists. The blue lines represent a quantile regression with 5 and 95% confidence limits. Note that the y-axis is the same among species.

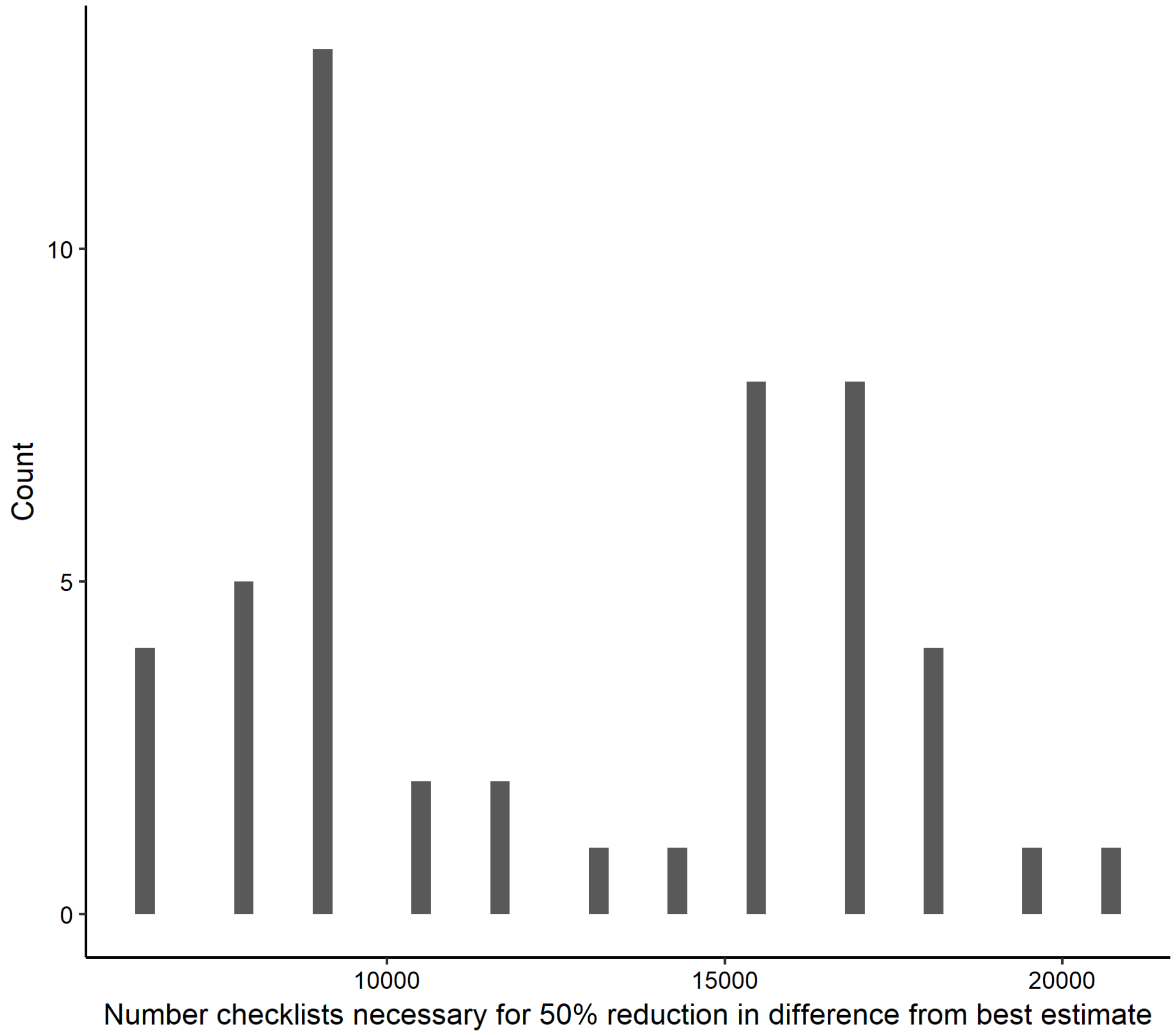

Fig S4. The number of checklists necessary for a 50% reduction in the absolute difference from the best estimate (Figs. S3a – S3e), showing that there is variation among species, but the median was ~11,700, with a min of ~ 6,500 and a max of ~ 20,800.

Appendix 3. Correlation plots showing the relationships between the predictor variables for each of the grain sizes.

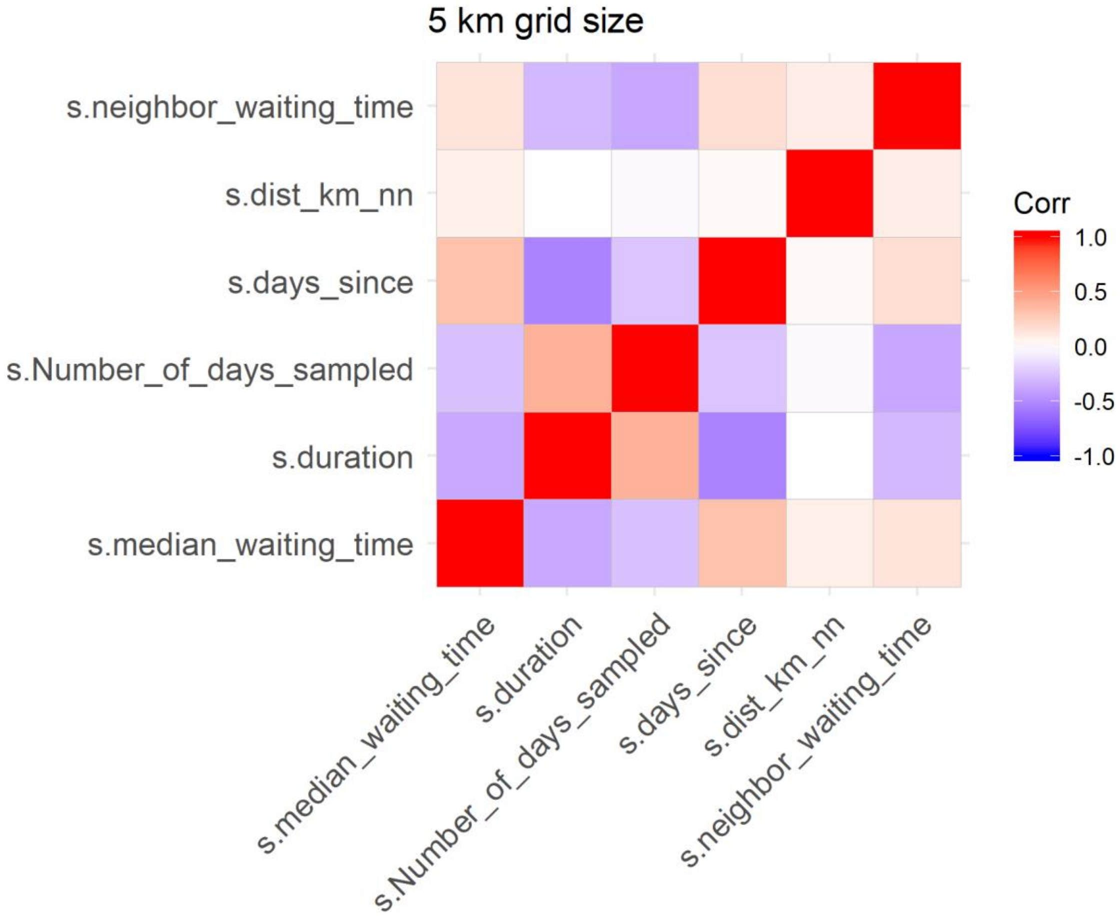

Fig. S5. Correlation plot showing the relationships between each of the initial potential predictor variables, standardized, for the 5 km grid cell size. Duration was highly correlated with median sampling interval for the majority of grid cell sizes, and as such, was excluded from consideration. s.neighbor_waiting_time is the nearest neighbor sampling interval; s.dist_km_nn is the distance to the nearest sampled grid; s.days_since is the number of days since the last sample; s.Number_of_days_samples is the number of unique days sampled; s.duration is the total duration between the original sample and the latest sample; s.median_waiting_time is the median sampling interval.

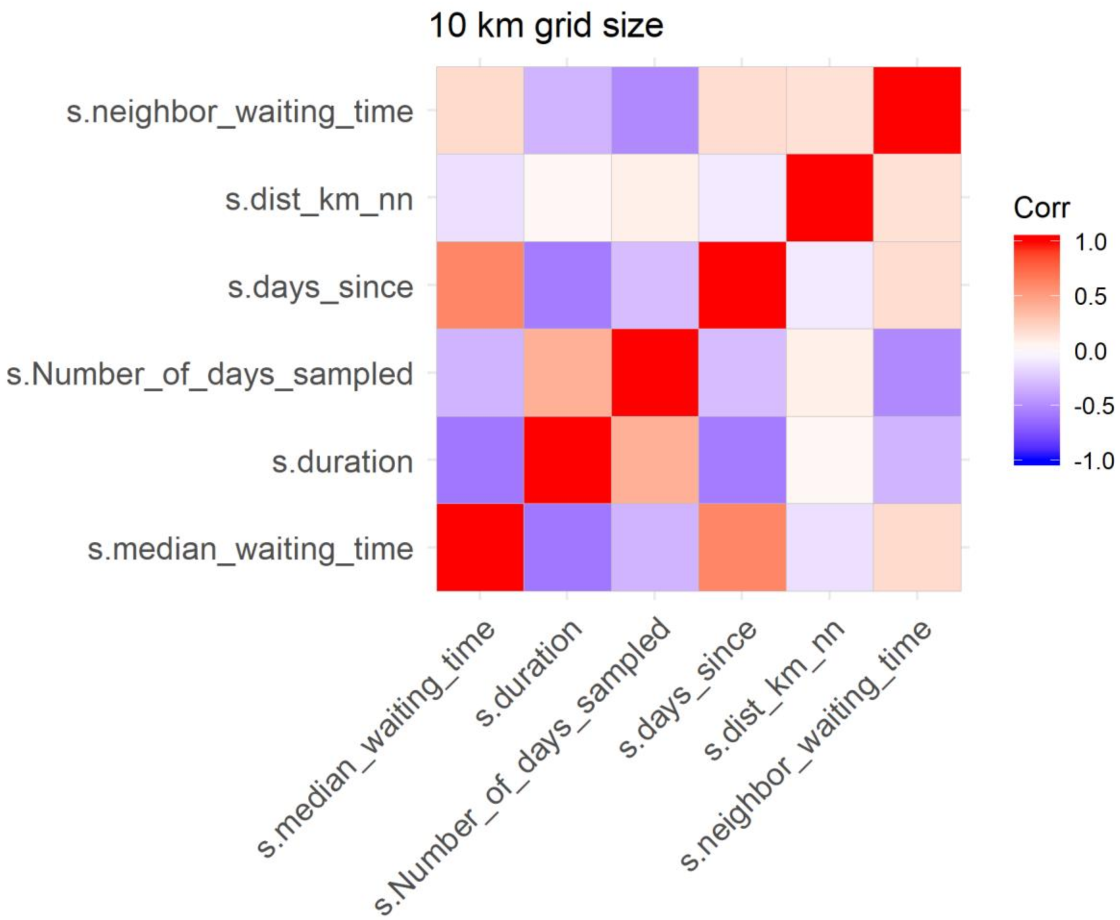

Fig. S6. Correlation plot showing the relationships between each of the initial potential predictor variables, standardized, for the 10 km grid cell size. Duration was highly correlated with median sampling interval for the majority of grid cell sizes, and as such, was excluded from consideration. s.neighbor_waiting_time is the nearest neighbor sampling interval; s.dist_km_nn is the distance to the nearest sampled grid; s.days_since is the number of days since the last sample; s.Number_of_days_samples is the number of unique days sampled; s.duration is the total duration between the original sample and the latest sample; s.median_waiting_time is the median sampling interval.

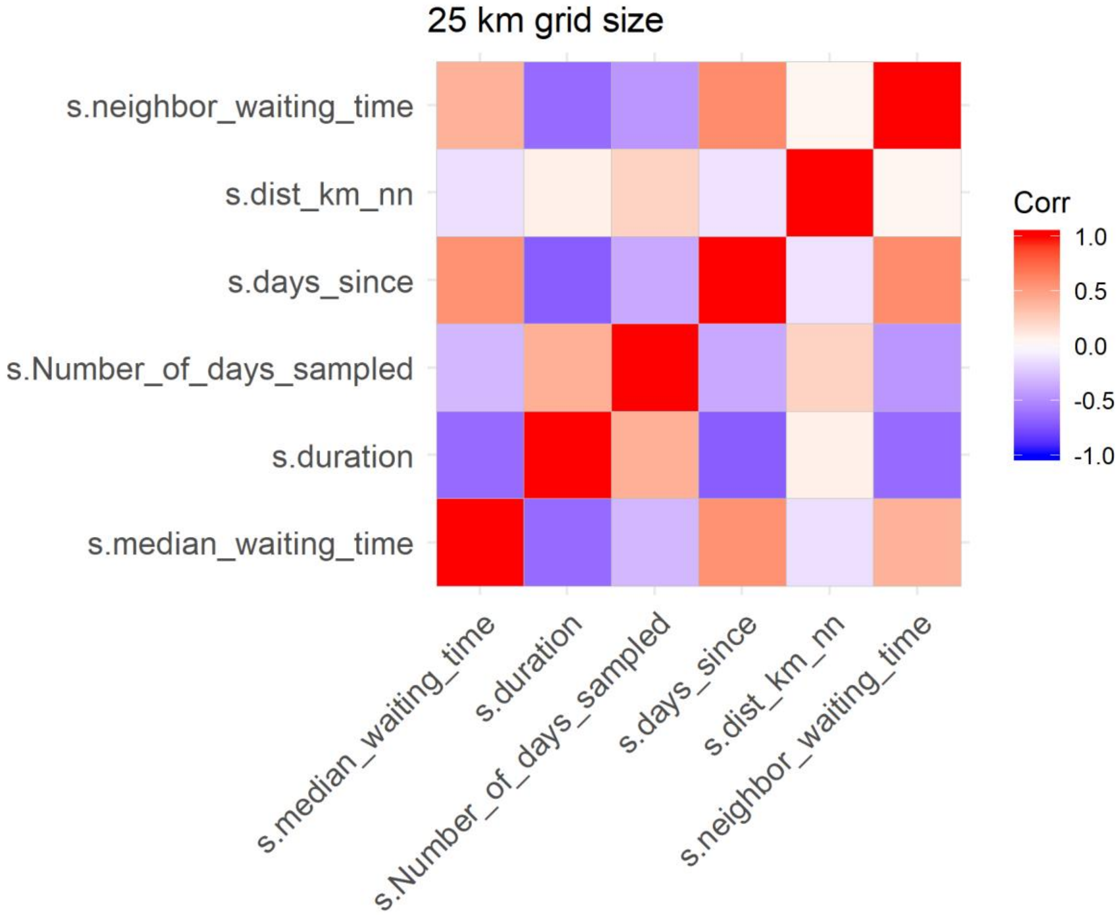

Fig. S7. Correlation plot showing the relationships between each of the initial potential predictor variables, standardized, for the 25 km grid cell size. Duration was highly correlated with median sampling interval for the majority of grid cell sizes, and as such, was excluded from consideration. s.neighbor_waiting_time is the nearest neighbor sampling interval; s.dist_km_nn is the distance to the nearest sampled grid; s.days_since is the number of days since the last sample; s.Number_of_days_samples is the number of unique days sampled; s.duration is the total duration between the original sample and the latest sample; s.median_waiting_time is the median sampling interval.

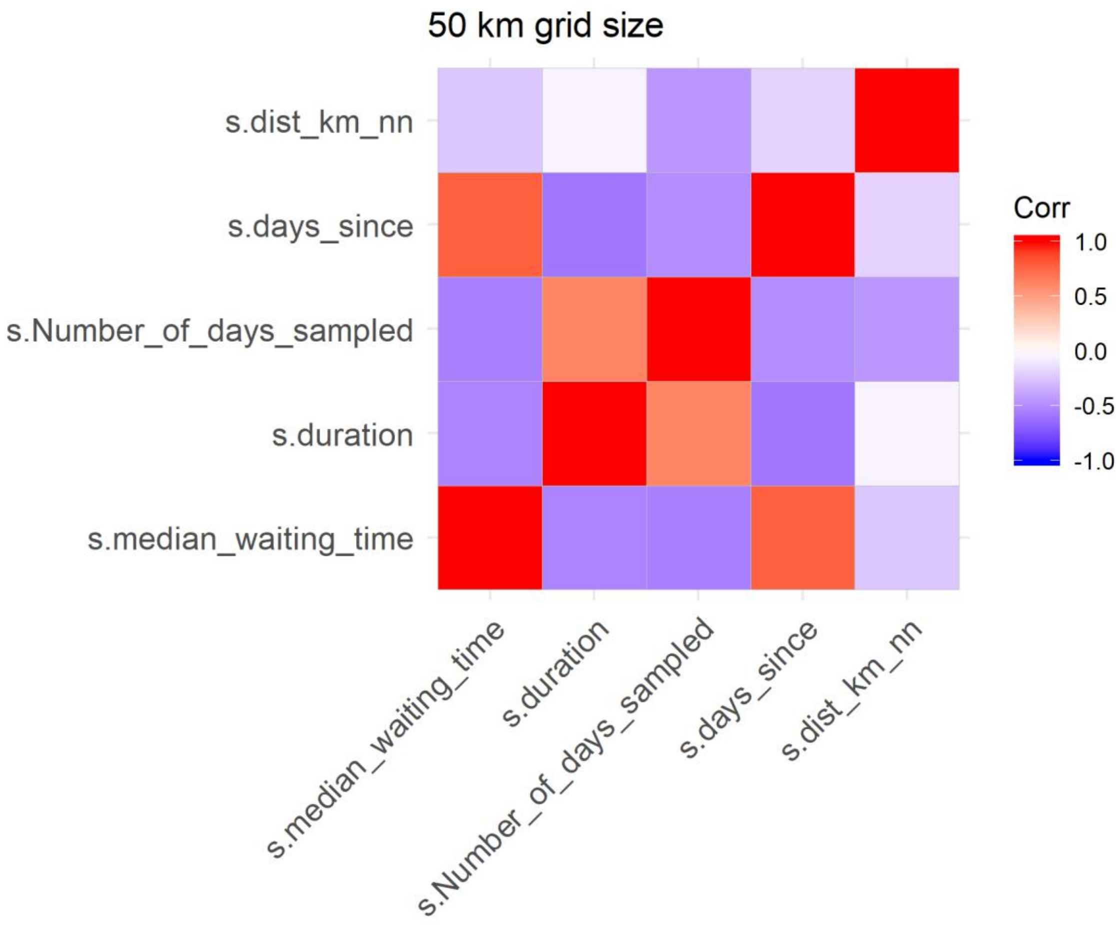

Fig. S8. Correlation plot showing the relationships between each of the initial potential predictor variables, standardized, for the 50 km grid cell size. Duration was highly correlated with median sampling interval for the majority of grid cell sizes, and as such, was excluded from consideration. s.neighbor_waiting_time is the nearest neighbor sampling interval; s.dist_km_nn is the distance to the nearest sampled grid; s.days_since is the number of days since the last sample; s.Number_of_days_samples is the number of unique days sampled; s.duration is the total duration between the original sample and the latest sample; s.median_waiting_time is the median sampling interval.

Appendix 4. Summary statistics for the daily-sampled parameters (i.e., predictors) for each of the four grain sizes (5, 10, 25, 50 km^2, respectively). Each parameter was calculated for each day in 2018: N=365 times, whereby only the preceding days in the year factored into the calculation and not the following days. Summaries were produces using the 'dfSummary' function from the summarytools package in R (https://cran.r-project.org/web/packages/summarytools/vignettes/Introduction.html).

Data Frame Summary

grid_5

Dimensions: 220791 x 7
Duplicates: 87137

No	Variable	Stats / Values	Freqs (% of Valid)	Graph	Missing
1	grid_id<br>[integer]	Mean (sd) : 294.5 (169.5)<br>min < med < max:<br>1 < 292 < 591<br>IQR (CV) : 292 (0.6)	591 distinct values	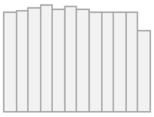	0<br>(0%)
2	sampled_or_not<br>[character]	1. no<br>2. yes	82783 (37.5%)<br>138008 (62.5%)	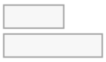	0<br>(0%)
3	median_sampling_interval<br>[numeric]	Mean (sd) : 149.1 (294.7)<br>min < med < max:<br>1 < 31 < 2450<br>IQR (CV) : 124 (2)	396 distinct values	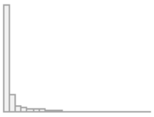	97125<br>(43.99%)
4	number_of_unique_days_sampled<br>[integer]	Mean (sd) : 100 (184.8)<br>min < med < max:<br>1 < 25 < 1222<br>IQR (CV) : 95 (1.8)	1222 distinct values	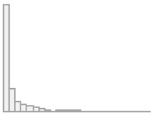	82783<br>(37.49%)
5	days_since_last_sample<br>[numeric]	Mean (sd) : 216.7 (406.1)<br>min < med < max:<br>1 < 39 < 2935<br>IQR (CV) : 228 (1.9)	2858 distinct values	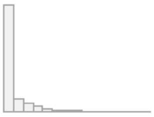	82783<br>(37.49%)
6	distance_to_nearest_sampled_grid_km<br>[numeric]	Mean (sd) : 6.4 (3.3)<br>min < med < max:<br>1.9 < 5 < 19.7<br>IQR (CV) : 2.1 (0.5)	674 distinct values	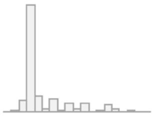	0<br>(0%)
7	nearest_neighbor_sampling_interval<br>[numeric]	Mean (sd) : 174.6 (302)<br>min < med < max:<br>1 < 43.5 < 2450<br>IQR (CV) : 170 (1.7)	367 distinct values	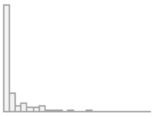	40819<br>(18.49%)

Generated by summarytools 0.9.4 (R version 3.5.0)
2019-08-26

Data Frame Summary

grid_10

Dimensions: 67681 x 7

Duplicates: 21389

No	Variable	Stats / Values	Freqs (% of Valid)	Graph	Missing
1	grid_id<br>[integer]	Mean (sd) : 81.6 (46.3)<br>min < med < max:<br>1 < 79 < 166<br>IQR (CV) : 78 (0.6)	166 distinct values	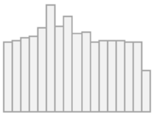	0<br>(0%)
2	sampled_or_not<br>[character]	1. no<br>2. yes	14438 (21.3%)<br>53243 (78.7%)	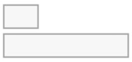	0<br>(0%)
3	median_sampling_interval<br>[numeric]	Mean (sd) : 96.2 (208.9)<br>min < med < max:<br>1 < 11 < 1401<br>IQR (CV) : 57 (2.2)	210 distinct values	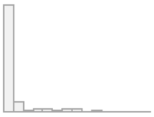	17746<br>(26.22%)
4	number_of_unique_days_sampled<br>[integer]	Mean (sd) : 303.7 (441.1)<br>min < med < max:<br>1 < 79 < 1894<br>IQR (CV) : 424 (1.5)	1885 distinct values	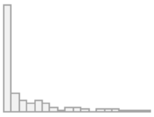	14438<br>(21.33%)
5	days_since_last_sample<br>[numeric]	Mean (sd) : 124 (256.7)<br>min < med < max:<br>1 < 10 < 1645<br>IQR (CV) : 86 (2.1)	1635 distinct values	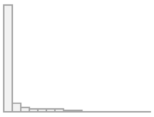	14438<br>(21.33%)
6	distance_to_nearest_sampled_grid_km<br>[numeric]	Mean (sd) : 9.8 (2.2)<br>min < med < max:<br>0 < 10 < 23.1<br>IQR (CV) : 0.7 (0.2)	179 distinct values	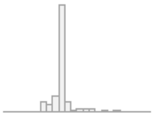	0<br>(0%)
7	nearest_neighbor_sampling_interval<br>[numeric]	Mean (sd) : 102.8 (209.7)<br>min < med < max:<br>1 < 14 < 1401<br>IQR (CV) : 54 (2)	177 distinct values	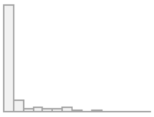	7058<br>(10.43%)

Generated by summarytools 0.9.4 (R version 3.5.0)
2019-08-26

Data Frame Summary

grid_25

Dimensions: 21992 x 7

Duplicates: 11273

No	Variable	Stats / Values	Freqs (% of Valid)	Graph	Missing
1	grid_id<br>[integer]	Mean (sd) : 14.8 (7.5)<br>min < med < max:<br>1 < 14 < 32<br>IQR (CV) : 9 (0.5)	32 distinct values	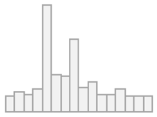	0<br>(0%)
2	sampled_or_not<br>[character]	1. no<br>2. yes	969 (4.4%)<br>21023 (95.6%)	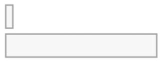	0<br>(0%)
3	median_sampling_interval<br>[numeric]	Mean (sd) : 31.1 (113.7)<br>min < med < max:<br>1 < 1 < 821<br>IQR (CV) : 5 (3.7)	66 distinct values	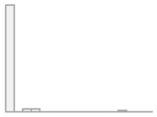	1018<br>(4.63%)
4	number_of_unique_days_sampled<br>[integer]	Mean (sd) : 1214.9 (1001.7)<br>min < med < max:<br>1 < 1103 < 2946<br>IQR (CV) : 2071.5 (0.8)	2099 distinct values	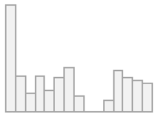	969<br>(4.41%)
5	days_since_last_sample<br>[numeric]	Mean (sd) : 26.9 (83.6)<br>min < med < max:<br>1 < 1 < 693<br>IQR (CV) : 4 (3.1)	601 distinct values	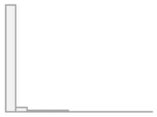	969<br>(4.41%)
6	distance_to_nearest_sampled_grid_km<br>[numeric]	Mean (sd) : 22 (2.7)<br>min < med < max:<br>14.8 < 22 < 25<br>IQR (CV) : 4.3 (0.1)	32 distinct values	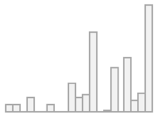	0<br>(0%)
7	nearest_neighbor_sampling_interval<br>[numeric]	Mean (sd) : 24.1 (104.9)<br>min < med < max:<br>1 < 2 < 821<br>IQR (CV) : 5 (4.4)	39 distinct values	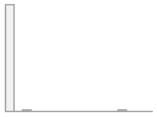	65<br>(0.3%)

Generated by summarytools 0.9.4 (R version 3.5.0)
2019-08-26

Data Frame Summary

grid_50

Dimensions: 15499 x 7

Duplicates: 12213

No	Variable	Stats / Values	Freqs (% of Valid)	Graph	Valid	Missing
1	grid_id<br>[integer]	Mean (sd) : 4.6 (2.1)<br>min < med < max:<br>1 < 4 < 10<br>IQR (CV) : 3 (0.5)	1 : 387 (2.5%)<br>2 : 1755 (11.3%)<br>3 : 3977 (25.7%)<br>4 : 1675 (10.8%)<br>5 : 2359 (15.2%)<br>6 : 3423 (22.1%)<br>7 : 365 (2.4%)<br>8 : 410 (2.6%)<br>9 : 783 (5.1%)<br>10 : 365 (2.4%)	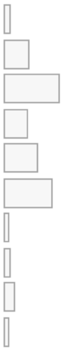	15499<br>(100%)	0<br>(0%)
2	sampled_or_not<br>[character]	1. no<br>2. yes	365 (2.4%)<br>15134 (97.7%)	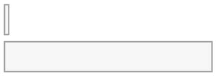	15499<br>(100%)	0<br>(0%)
3	median_sampling_interval<br>[numeric]	Mean (sd) : 6.3 (26.3)<br>min < med < max:<br>1 < 1 < 193<br>IQR (CV) : 0 (4.2)	20 distinct values	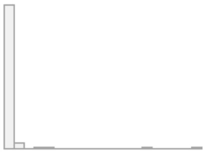	15134<br>(97.65%)	365<br>(2.35%)
4	number_of_unique_days_sampled<br>[integer]	Mean (sd) : 2062 (743.7)<br>min < med < max:<br>11 < 2284 < 3004<br>IQR (CV) : 808 (0.4)	1553 distinct values	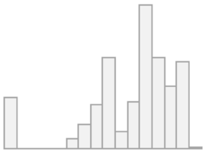	15134<br>(97.65%)	365<br>(2.35%)
5	days_since_last_sample<br>[numeric]	Mean (sd) : 6.2 (28.7)<br>min < med < max:<br>1 < 1 < 316<br>IQR (CV) : 0 (4.7)	316 distinct values	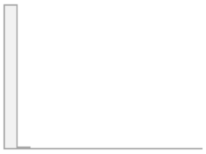	15134<br>(97.65%)	365<br>(2.35%)
6	distance_to_nearest_sampled_grid_km<br>[numeric]	Mean (sd) : 38.9 (3.2)<br>min < med < max:<br>32.3 < 37.5 < 45.1<br>IQR (CV) : 3.6 (0.1)	32.34! : 365 (2.4%)<br>35.74! : 3977 (25.7%)<br>37.53! : 3423 (22.1%)<br>38.07! : 365 (2.4%)<br>39.35! : 4034 (26.0%)<br>42.36! : 387 (2.5%)<br>43.10! : 783 (5.1%)<br>44.79! : 1755 (11.3%)<br>45.13! : 410 (2.6%)<br>! rounded	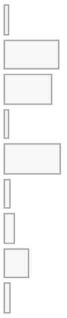	15499<br>(100%)	0<br>(0%)
7	nearest_neighbor_sampling_interval<br>[numeric]	1 distinct value	1 : 15499 (100.0%)	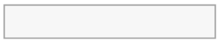	15499<br>(100%)	0<br>(0%)

Generated by summarytools 0.9.4 (R version 3.5.0)
2019-08-26

Appendix 5. Distribution of sampling within 5 km grids throughout 15 regional cities in Australia.

In order to investigate the generalizability of our results to other regions in Australia, we investigated the distribution of samples per grid for 5 km grids among 15 regional cities throughout Australia. We extracted coordinates for each city from: https://latitudelongitude.org. We then extracted all eBird checklists which met our criteria in the main manuscript within a 50 km buffer of each of these regional city's coordinates. This approach slightly differed to our analysis presented in the main text because we had a shapefile of the Greater Sydney Region, but we did not have access to such shapefiles for other cities. We then gridded each region into 5 km grids, as in the main manuscript, and calculated the number of eBird checklists per each grid. Because the distributions below (Fig. S9) are very similar – many unsampled or poorly-sampled grids in most cities with a similar distribution aside from these (e.g., few well-sampled grids) – we believe that each region would be at the same 'starting point'. In other words, the current sampling regimes among regions are all very similar – especially in regions where the cities receive similar sampling intensities (e.g., Brisbane, Melbourne, Sydney). But some remote regions receive relatively few eBird checklists (e.g., Longreach).

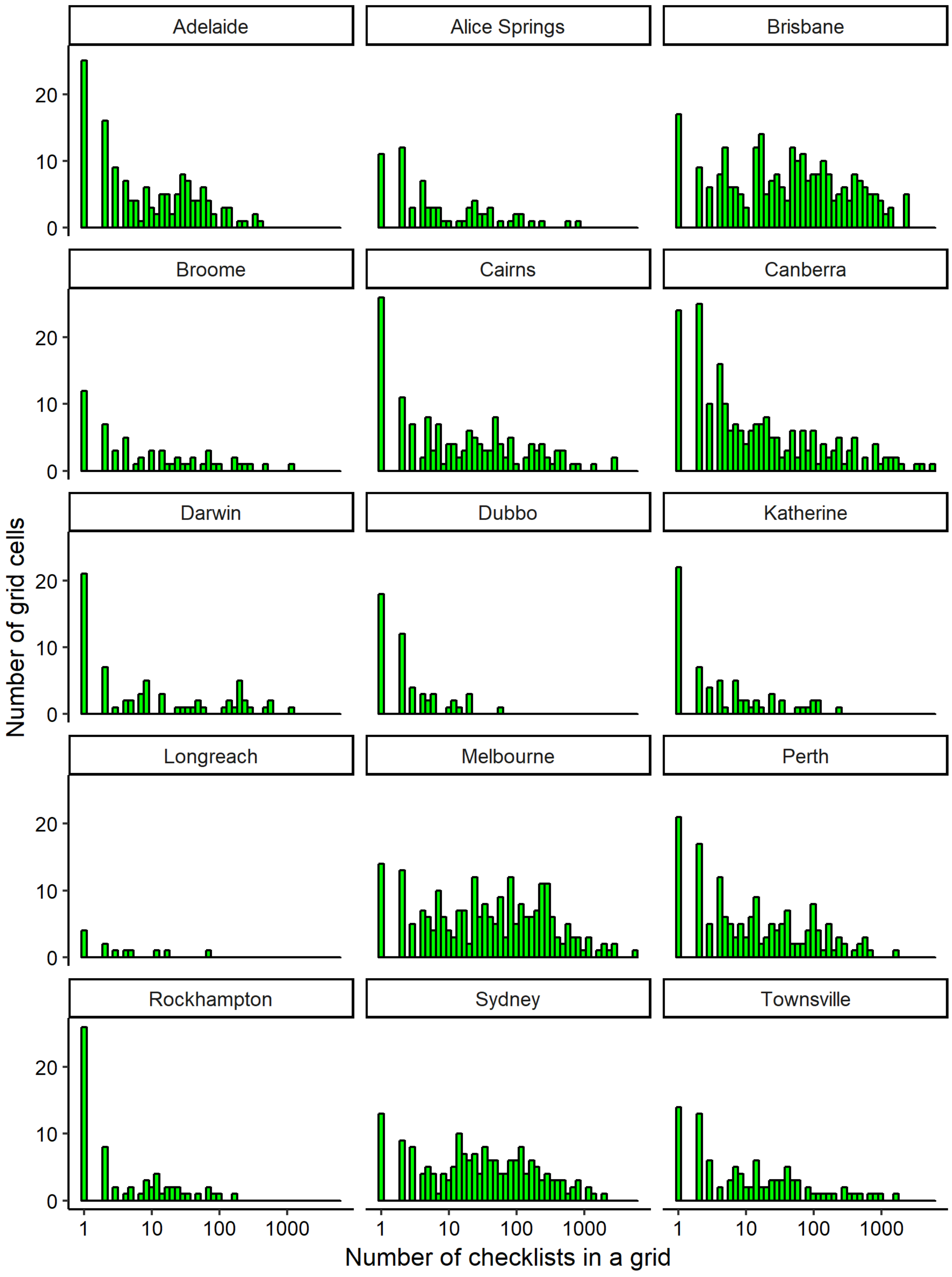

Fig. S9. The distribution of number of checklists within a grid for each of 15 regional cities in Australia, showing a similar distribution among regions (e.g., many unsampled or poorly sampled grids and then variation among the better-sampled grids).
